# Environmental Conditions Affect Exhalation of H3N2 Seasonal and Variant Influenza Viruses and Respiratory Droplet Transmission in Ferrets

**DOI:** 10.1371/journal.pone.0125874

**Published:** 2015-05-13

**Authors:** Kortney M. Gustin, Jessica A. Belser, Vic Veguilla, Hui Zeng, Jacqueline M. Katz, Terrence M. Tumpey, Taronna R. Maines

**Affiliations:** Influenza Division, National Center for Immunization and Respiratory Disease, Centers for Disease Control and Prevention, Atlanta, Georgia, United States of America; Virginia Polytechnic Institute and State University, UNITED STATES

## Abstract

The seasonality of influenza virus infections in temperate climates and the role of environmental conditions like temperature and humidity in the transmission of influenza virus through the air are not well understood. Using ferrets housed at four different environmental conditions, we evaluated the respiratory droplet transmission of two influenza viruses (a seasonal H3N2 virus and an H3N2 variant virus, the etiologic virus of a swine to human summertime infection) and concurrently characterized the aerosol shedding profiles of infected animals. Comparisons were made among the different temperature and humidity conditions and between the two viruses to determine if the H3N2 variant virus exhibited enhanced capabilities that may have contributed to the infections occurring in the summer. We report here that although increased levels of H3N2 variant virus were found in ferret nasal wash and exhaled aerosol samples compared to the seasonal H3N2 virus, enhanced respiratory droplet transmission was not observed under any of the environmental settings. However, overall environmental conditions were shown to modulate the frequency of influenza virus transmission through the air. Transmission occurred most frequently at 23°C/30%RH, while the levels of infectious virus in aerosols exhaled by infected ferrets agree with these results. Improving our understanding of how environmental conditions affect influenza virus infectivity and transmission may reveal ways to better protect the public against influenza virus infections.

## Introduction

Influenza viruses display distinct seasonal patterns in temperate climates with peak infection rates occurring in the winter months and little to no influenza virus activity detected at other times of the year [1]. However, it is not completely understood why we have “flu seasons”. The same seasonal trends do not necessarily apply to regions in tropic and subtropic climates where influenza virus activity can be detected year round. Social and health factors such as indoor crowding and seasonal fluctuations in host immunity have been offered as explanations for influenza seasonality, but the lack of direct evidence supporting these theories suggests that the environment may be the most relevant contributor and potential driving force of seasonality [1,2]. Although seasonal influenza viruses display predictable infection patterns, the four most recent influenza virus pandemics began with newly emerged viruses appearing outside the usual influenza season (1918 Spanish Influenza A H1N1, 1957–1958 Asian Influenza A H2N2, 1968 Hong Kong Influenza A H3N2, 2009 H1N1) [3,4]. Emergence of the most recent pandemic virus was preceded by reports of sporadic infections of a swine-origin H1N1 virus in young individuals [4,5]. During the summers of 2010–2014, reports have emerged in the Midwest region of the U.S. of H3N2 variant influenza virus infections in children with a recent history of exposure to swine [6–10]. These events highlight the importance of closely monitoring emerging strains of influenza viruses throughout the year and the need for our improved understanding of the effects of environmental conditions like temperature and humidity on influenza virus transmission.

There are three generally accepted modes of influenza virus transmission [11]. Aerosol transmission occurs via respirable particles (<5 μm) that may remain suspended in the air for prolonged periods of time thus increasing the opportunity for them to be inhaled. Droplet transmission occurs when larger droplets (>5 μm), typically expelled during coughing or sneezing, encounter the upper respiratory tract or ocular mucosa of a susceptible individual. Lastly, contact transmission involves directly touching an infected host or contaminated surface. The relative contribution of these modes of transmission to the overall spread of influenza viruses and the role of environmental conditions is not completely understood. However, recent studies in human volunteers support a role for aerosols in influenza virus transmission [12,13].

Temperature and humidity are inextricably linked when considering their effects on evaporation rates and on infectious particle dynamics [14]. Although the majority of reports of increased virus viability in low humidity have been focused on relative humidity (RH), it has been demonstrated that there is also a relationship between influenza virus seasonality and absolute humidity (AH) [15]. RH is the ratio of water vapor in the air to the maximum amount the air can hold at a particular temperature. AH represents the mass of water vapor present within a unit of air [1,15]. Previous *in vitro* studies have demonstrated that influenza viruses in droplets and aerosols maintain viability better at low humidity [16,17]. Animal models have also been used to address the role of environmental conditions on influenza virus transmission and have shown that low temperature and RH environments support transmission among guinea pigs [18]. The ferret model has become an invaluable tool for evaluating virulence and transmissibility of influenza viruses and is a critical component of risk assessments performed on emerging influenza viruses that pose a risk to public health [19]. To more clearly understand the influence of temperature and humidity on influenza virus in exhaled aerosols and on transmission, we housed ferrets under four distinct environmental conditions and evaluated the respiratory droplet (RD) transmission of two highly transmissible H3N2 influenza viruses: A/Panama/2007/1999 (PN99), a seasonal H3N2 virus, and A/Indiana/8/2011 (IN11), an H3N2 variant (H3N2v), triple-reassortant swine influenza virus containing the 2009 pandemic H1N1 M gene that was isolated from a human in the summer of 2011 [7]. During these transmission experiments, the aerosol particle size distribution and the levels of infectious virus exhaled from infected ferrets were also assessed. Here, we demonstrate that the amount of influenza virus exhaled by infected ferrets and, ultimately, RD transmission are influenced by the temperature and humidity in which the animals are housed. These findings draw attention to the importance of climatic effects on influenza virus transmission and the potential for temperature and humidity to play a role in the emergence of influenza viruses that pose a risk to public health.

## Materials and Methods

### Ethics Statement

All ferret procedures were approved by Institutional Animal Care and Use Committee (IACUC) of the Centers for Disease Control and Prevention and in an Association for Assessment and Accreditation of Laboratory Animal Care International-accredited facility. Animal studies were performed in accordance with the IACUC guidelines under protocol #2234MAIFERC: "Transmissibility of influenza viruses with pandemic potential".

### Viruses

Virus stocks of A/Panama/2007/1999 (H3N2) were propagated in the allantoic cavity of 10 day old embryonated hens’ eggs while A/Indiana/8/2011 (H3N2v) was grown in Madin-Darby canine kidney cells (MDCK) as previously described [20]. Titers of virus stocks were determined by standard plaque assay using MDCK cells and are reported as plaque-forming units (pfu)/mL.

### Ferret Housing Conditions and Inoculations

Male Fitch ferrets 5–11 months of age (Triple F Farms), serologically negative by hemagglutination-inhibition (HI) assay for currently circulating influenza viruses, were housed during each experiment in cages placed inside a custom environmental chamber (Bahnson Environmental Specialties, Raleigh, NC) with HEPA filtration operating at 20 air changes per hour. Experiments were conducted at four environmental settings with consideration for the comfort of the animals: 5°C/70%RH (4.8 g/m^3^ AH); 23°C/30%RH (6.2 g/m^3^ AH); 23°C/50%RH (10.3 g/m^3^ AH) and 23°C/70%RH (14.4 g/m^3^ AH). Prior to inoculation, ferrets were allowed to acclimatize inside the chamber for at least four days. Baseline temperatures were measured using an implantable subcutaneous temperature transponder (BioMedic Data Systems, Seaford, DE) and baseline serum, weight and minute volume (MV) of respiration measurements were collected as described [20,21].

For each virus and each condition, three ferrets were sedated with a ketamine/zylazine/atropine hydrochloride cocktail injection and were presented with 10^3.8^ to 10^5.5^ pfu of aerosolized virus as described [21] using an aerosol exposure system with an AeroMP management platform (Biaera Technologies, Hagerstown, MD). Inoculations were performed at ambient laboratory temperatures (21±1°C) and at the corresponding experimental RH. Aerosol presented dose (PD) refers to the amount of virus inhaled by the animal, not necessarily the amount that is deposited within the respiratory tract. PD is based on the time of exposure, baseline MV of respiration of the animal and the aerosolized virus concentrations achieved during the exposure session.

### Ferret Transmission Experiments

RD transmission was assessed for each virus at each of four environmental conditions. Experiments were conducted using animal cages modified with perforated side walls which allow transmission of virus through the air while preventing direct or indirect contact between inoculated and naïve ferrets. Airflow velocity was measured inside the cages housed within the chamber using a hot wire anemometer with a telescopic probe (General Tools, New York, NY) and was found to be negligible (<0.05 m/s) for all of the cages. One day post inoculation (dpi) a naïve ferret was placed in a cage adjacent to each inoculated animal. Nasal washes (NW) were collected every other day for up to 11 dpi or days post contact (dpc) [22] for virus titration by standard plaque assay in MDCK cells [23]. Clinical signs of infection including weight loss, fever and activity levels [22] were recorded daily for inoculated and contact animals. Sneezing frequency in animals was not audible from inside the chamber and therefore was not recorded. MV of respiration was measured on 2, 4 and 6 dpi using whole-body plethysmography (Buxco) in inoculated animals. At the experimental endpoint, ferrets were sedated and euthanatized by injection of a euthanasia agent containing pentobarbital. Transmission was confirmed by seroconversion via hemagglutination inhibition assay using sera collected from animals at least 18 days after exposure.

### Analysis of Aerosols

Three inoculated ferrets from each experiment were anesthetized and exhaled aerosol samples were collected and analyzed for size distribution using an aerodynamic particle sizer (APS; TSI Inc, Shoreview, MN). Aerosol samples were collected from ferrets immediately after exhalation on 2, 4 and 6 dpi for 15 minutes of closed-mouth, normal breathing and for 5 minutes of induced sneezing as described [24]. Size distribution data represent the total particle counts or volumes collected for aerosols with aerodynamic diameters in the range of 0.5–20 μm. Volume data were derived using the DistFit software package (Chimera Technologies).

Exhaled aerosols from inoculated animals were analyzed for infectious virus using a viable two-stage cascade impactor (Tisch Environmental, Cleves, OH) on 1, 3, and 5 dpi as described with modifications [24]. Aerosols were collected from ferrets immediately after exhalation for 15 minutes of normal breathing and 5 minutes of sneezing and separated onto two stages by size (>4.7 μm or 0.65–4.7 μm). Aerosol samples were immediately processed by plaque assay without dilution and RNA extraction for real-time RT-PCR as described [24]. Influenza virus M gene RNA copy numbers were extrapolated using a standard curve based on samples of known virus titer (pfu/mL).

The effects of the aerosol collection procedure on influenza virus recovery was evaluated in duplicate by applying a known amount of virus (10^2^–10^3^ pfu) directly on prepared impactor plates and placing the impactor assembly inside the environmental chamber at one of the four environmental conditions [24]. Using a vacuum, air was then pulled through the impactor for 15 minutes or 5 minutes to reflect the collection times for normal breathing or sneezing, respectively. The aerosol collection media was harvested and processed as described for ferret sample collection to determine the percentage of RNA and infectious virus recovered. These recovery rates were used to estimate the amount of virus exhaled by ferrets at all of the time points in which infectious virus was detected.

### Statistics

Linear mixed models were used to estimate the means and differences in means for weight loss and aerosol volume data. Square root transformation of the aerosol volume data was needed to normalize the values. Further, because the weight loss data at any given time point was dependent on the previous time point data collection, a repeated measures model was utilized to estimate the respective means. To best determine the relationship between mean weight loss and ferret housing condition, the following independent variables were included in the model: ferret housing condition and day of weight measurement. For models associated with the aerosol volume data, the independent parameters included were: virus, ferret housing condition, and exhaled aerosol sample size. An interaction term was also included in the model to evaluate whether aerosol volume data varied based on virus, ferret housing condition, and exhaled aerosol sample size. Lastly, a Poisson regression model was used to estimate the means and differences in means for aerosol virus count data. The independent variables in the Poisson models included: virus, ferret housing condition, exhaled aerosol sample size and interaction terms. The interaction terms between independent variables were developed so as to allow for the testing of mean differences between groups or conditions. A p-value of <0.05 was considered significant. Analyses were performed using SAS software version 9.3 (SAS Institute Inc., Cary, NC).

## Results

### Morbidity observed in ferrets housed under diverse environmental conditions

During RD transmission experiments, six ferrets were housed in an environmental chamber set to one of four experimental conditions (5°C/70%RH, 23°C/30%RH, 23°C/50%RH, or 23°C/70%RH) (Table 1) and allowed to acclimatize for at least four days prior to each experiment. The temperature and humidity settings were chosen to represent a range of conditions with consideration for the functional limitations of the chamber and the tolerance level of the animals. For each condition, three ferrets were inoculated by aerosol inhalation with 10^3.8^–10^5.5^ pfu of PN99 or IN11 virus (Table 1). One dpi, a naïve ferret was placed in an adjacent cage to an inoculated ferret, each cage with a perforated side wall, allowing for the transfer of respiratory droplets through the air while preventing any direct or indirect contact between the animal pairs. Inoculated and contact animals were monitored for clinical signs of infection for up to 11 dpi or dpc to establish differences in morbidity in ferrets housed under each environmental condition.

**Table 1:** Replication and respiratory droplet transmission of PN99 and IN11 influenza viruses in ferrets housed at different environmental conditions.

			Inoculated^a^					Contacts^b^		
	Environmental Condition (°C/%RH)	Absolute Humidity (g/m^3^)	Presented Dose (log_10_ pfu)	Peak Temp Change^e^ (day)^d^	Peak % Weight Loss (day)^d^	Peak NW^c^ Titer (day)^d^	RDT^f^ (day)	Peak Temp Change^e^ (day)^d^	Peak % Weight Loss (day)^d^	Peak NW^c^ Titer (day)^d^
PN99	5/70	4.8	3.8±0.10	1.2 (2)	16.5 (9)	4.7±0.30 (3)	1/3 (3)	0.9 (9)	8.7 (9)	4.4 (5)
	23/30	6.2	4.2±0.09	1.4 (2)	15.3 (7)	4.8±0.61 (3)	2/3 (3)	2.0 (5)	8.4 (7)	3.4±1.40 (5)
	23/50	10.3	4.3±0.13	1.8 (2)	9.6 (7)	4.2±0.48 (3)	1/3 (5)	0.7 (3)	5.2 (5)	3.8 (5)
	23/70	14.4	3.8±0.12	2.4 (2)	11.0 (7)	4.3±0.48 (5)	2/3 (3)	1.7 (5)	7.6 (9,11)	4.7±0.30 (3)
IN11	5/70	4.8	5.3±0.02	1.9 (3)	11.0 (9)	5.4±0.40 (3)	0/3	-	-	-
	23/30	6.2	4.3±0.18	3.3 (2)	10.7 (6)	5.3±0.56 (1)	3/3 (3)	0.4 (3)	3.7 (9)	5.3±0.58 (3)
	23/50	10.3	5.5±0.02	3.2 (2)	12.2 (7)	5.2±0.89 (5)	0/3	-	-	-
	23/70	14.4	4.8±0.14	2.0 (2)	13.4 (6)	5.7±0.26 (1)	2/3 (1)	2.1 (9)	2.1 (9)	4.8±1.10 (7)

^a^Data shown represent the mean of 3 inoculated animals.

^b^Data shown represent infected, contact animals only.

^c^NW, Nasal wash titer (log_10_ pfu/mL) ± SD is shown.

^d^Day post inoculation or post contact the peak was observed is shown in parentheses.

^e^Temperature change over baseline (35.7–39.6°C) is shown.

^f^RDT, Respiratory droplet transmission frequency and the day post-exposure that virus was first detected in contact animals is shown in parentheses.

**Fig 1:**
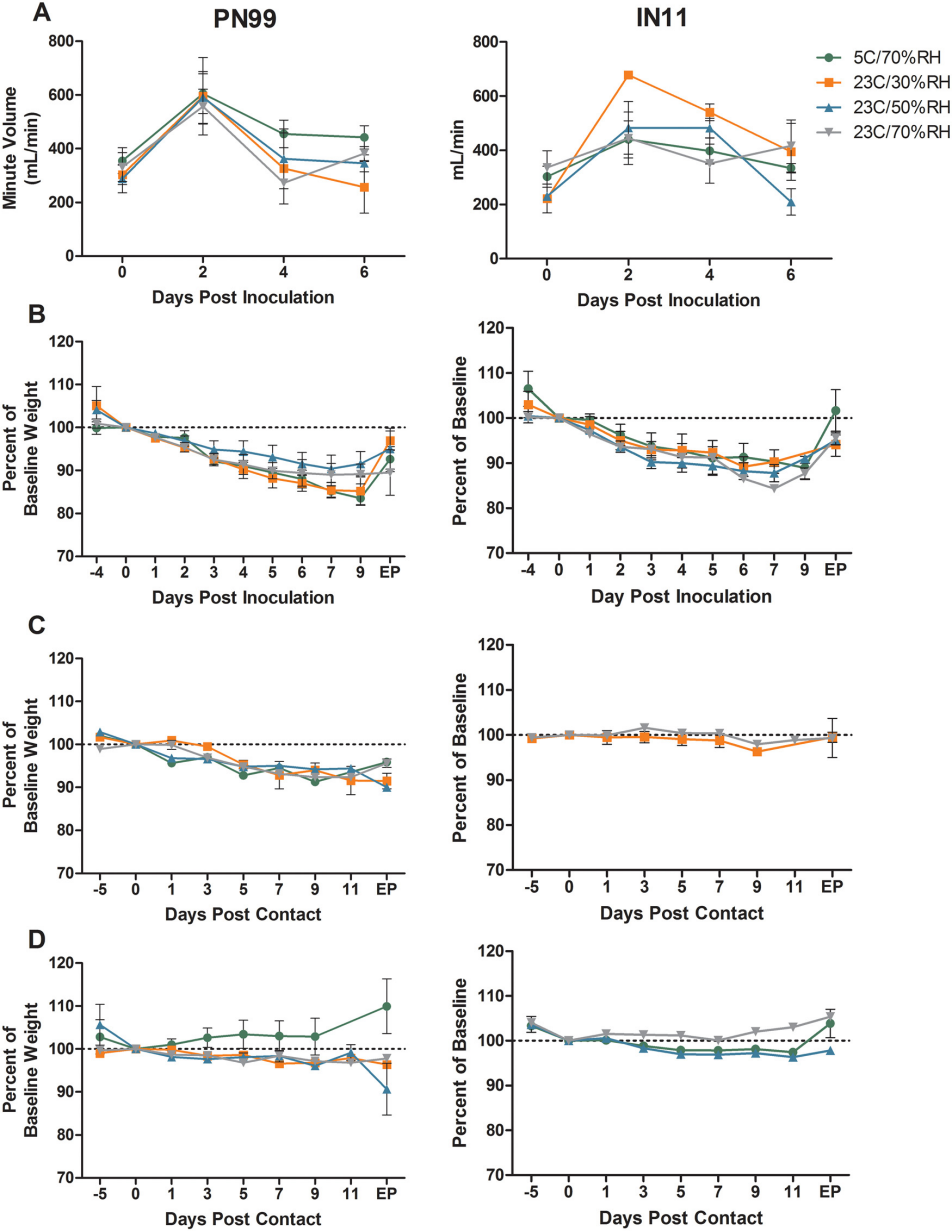
Morbidity observed in influenza virus infected and uninfected ferrets. Six ferrets each were housed under the designated environmental conditions for the purpose of respiratory droplet transmission experimentation. Three ferrets each were presented with 10^3.8^–10^5.5^ pfu of PN99 or IN11 virus by aerosol inhalation and contact ferrets, n = 3, were placed in adjacent cages one day later. Inoculated animals, n = 3, were monitored for changes in minute volume by plethysmography (A) and weight (B) on the days noted. Endpoint (EP) data were collected on days 19–29 post inoculation. Both infected (C) and uninfected (D) contact animals were monitored for weight loss. Any contact animal shedding detectable virus in nasal washes and sero-converting to homologous virus was considered infected. EP data were collected on days 18–28 post contact. Mean values ± SEM is shown.

Regardless of the inoculum, changes in activity levels in infected animals were minor (relative inactivity indexes ranged from 1.0–1.2) while anorexia and nasal discharge were the most common clinical signs observed in ferrets housed at any of the controlled environmental settings. Peak temperatures were detected 2–3 dpi in inoculated animals and ranged from 1.2–2.4°C above baseline for PN99 virus and 1.9–3.3°C above baseline for IN11 virus (Table 1). MV of respiration was measured by plethysmography at 0, 2, 4 and 6 dpi for each inoculated animal (Fig 1A). Respiration patterns were generally similar for PN99 and IN11 virus-inoculated ferrets housed at any of the environmental conditions with MV peaking 2 dpi for most of the animals and the largest change in respiration of ferrets in the IN11 virus group housed at 23°C/30%RH. Mean maximum weight loss in PN99 virus-inoculated ferrets was greatest (15.3–16.5%), although not significantly (p = 0.489), at the lower AH settings. Mean maximum weight loss was more similar across the environmental conditions for animals in the IN11 virus group, ranging from 10.7–13.4% (Table 1, Fig 1B). Weight loss observed in animals infected after exposure to inoculated animals (i.e. infected contacts) was not as severe, with mean maximum values ≤8.7% for all groups (Table 1, Fig 1C), presumably due to differences in dose received by these animals compared to inoculated ferrets. Furthermore, uninfected animals from the transmission experiments exhibited minimal weight loss regardless of housing condition (Fig 1D). Together these data show that while the housing conditions alone did not result in considerable weight loss in any of the animals, morbidity in inoculated animals may have been enhanced under certain conditions compared to those animals that were infected from exposure to inoculated ferrets.

### Influenza virus shedding kinetics in NW samples and RD transmission among ferrets

**Fig 2:**
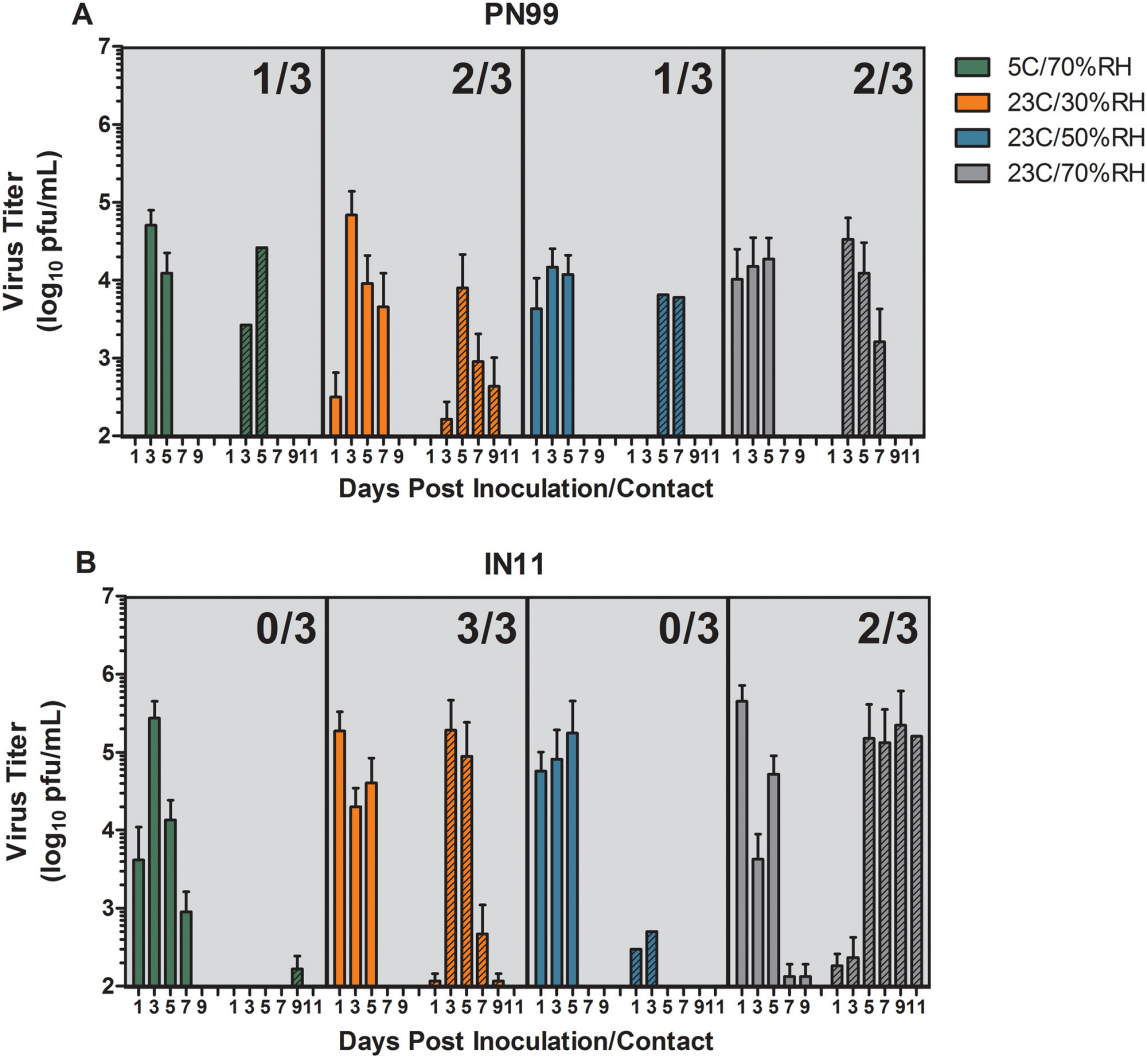
Respiratory droplet transmission of PN99 and IN11 influenza virus among ferrets. Three ferrets each were housed under the designated environmental conditions and were presented with 10^3.8^–10^5.5^ pfu of PN99 (A) or IN11 (B) virus by aerosol inhalation. One day later, a naïve, contact ferret was placed in a cage adjacent to each inoculated animal. Nasal washes were collected from ferrets every two days for up to 11 days and virus titers were assessed by plaque assay. Solid bars represent data from inoculated ferrets, n = 3, and hatched bars represent contact ferret data. The mean ± SD is shown and the limit of virus detection is 100pfu/mL. Frequency of transmission events is shown as an inset on each panel and represents the number of contact animals that shed virus and seroconverted.

RD transmission experiments were performed at each environmental setting to assess the ability of PN99 and IN11 viruses to transmit through the air among ferrets housed under different environmental conditions. Successful transmission was noted when virus was detected in nasal wash samples within 11 dpc and hemagglutination inhibition antibody titers against homologous virus were detected in convalescent serum collected after 18 dpc. Mean peak NW titers ranged from 10^4.2^ to 10^4.8^ pfu/mL in ferrets inoculated with PN99 virus and were 3–24 times higher (10^5.2^ to 10^5.7^ pfu/mL) in ferrets inoculated with IN11 virus housed under the respective environmental condition; the greatest difference was observed at the 23°C/70%RH condition (Table 1, Fig 2). Virus was first detected in NW samples from all inoculated animals by 1–3 dpi and was cleared by 7 dpi in all PN99 virus-inoculated animals, except for one ferret housed at 23°C/30%RH, and by 9 dpi in all but one ferret in the IN11 virus group housed at 23°C/70%RH (Fig 2). Overall, levels of virus shedding in NW samples were higher in the IN11 virus-inoculated animals compared to PN99 virus. Linear regression models using area under the curve values were used to determine if any of the environmental conditions served as a significant driving force for the observed differences in NW virus titers between the two viruses. Comparisons made between the virus groups was significant (p = 0.0004) but differences between the conditions was not (p = 0.4243) indicating that any observed differences between NW virus titers at any of the environmental conditions was related more to the virus than to the environmental condition itself.

Despite the higher titers in NW samples collected from IN11 virus inoculated ferrets, increased transmission was not observed compared to PN99 virus (Table 1, Fig 2). At 5°C/70%RH, transmission occurred in 1 of 3 ferrets exposed to PN99 virus-infected animals by 3 dpc and no transmission was observed in the IN11 virus contact group. Trace levels of IN11 virus were detected among some animals late in the course of infection but without seroconversion. The frequency of transmission events at 23°C/30%RH was greatest with 2 of 3 ferrets for PN99 virus by 3–5 dpc and 3 of 3 ferrets for IN11 virus by 3 dpc. One of the animals in the pair that did not transmit PN99 virus had an unknown underlying condition that required euthanasia at 7 dpi which may have affected transmission between this pair. At 23°C/50%RH, 1 of 3 PN99 virus-infected animals transmitted virus to a contact animal after 5 dpc while no transmission occurred in the IN11 virus group despite detection of baseline levels of virus 1–3 dpc but without seroconversion. It is not clear why productive infections did not occur in these animals. Transmission was detected by 3 dpc in 2 of 3 ferret pairs at 23°C/70%RH in both the PN99 and IN11 virus groups. Virus was cleared in all contact animals by 11 dpc except for a single ferret housed at 23°C/70%RH that had >10^5^ pfu/mL of IN11 virus in the NW sample for that day. Collectively, the frequency of RD transmission events was similar between the two viruses at any of the environmental conditions and transmission occurred least often among ferrets housed at 5°C/70%RH and 23°C/50%RH at 17%, while transmission frequency was highest at 83% under the 23°C/30%RH environmental conditions and was 67% at 23°C/70%RH.

### Analysis of aerosols exhaled by influenza virus-infected ferrets

**Fig 3:**
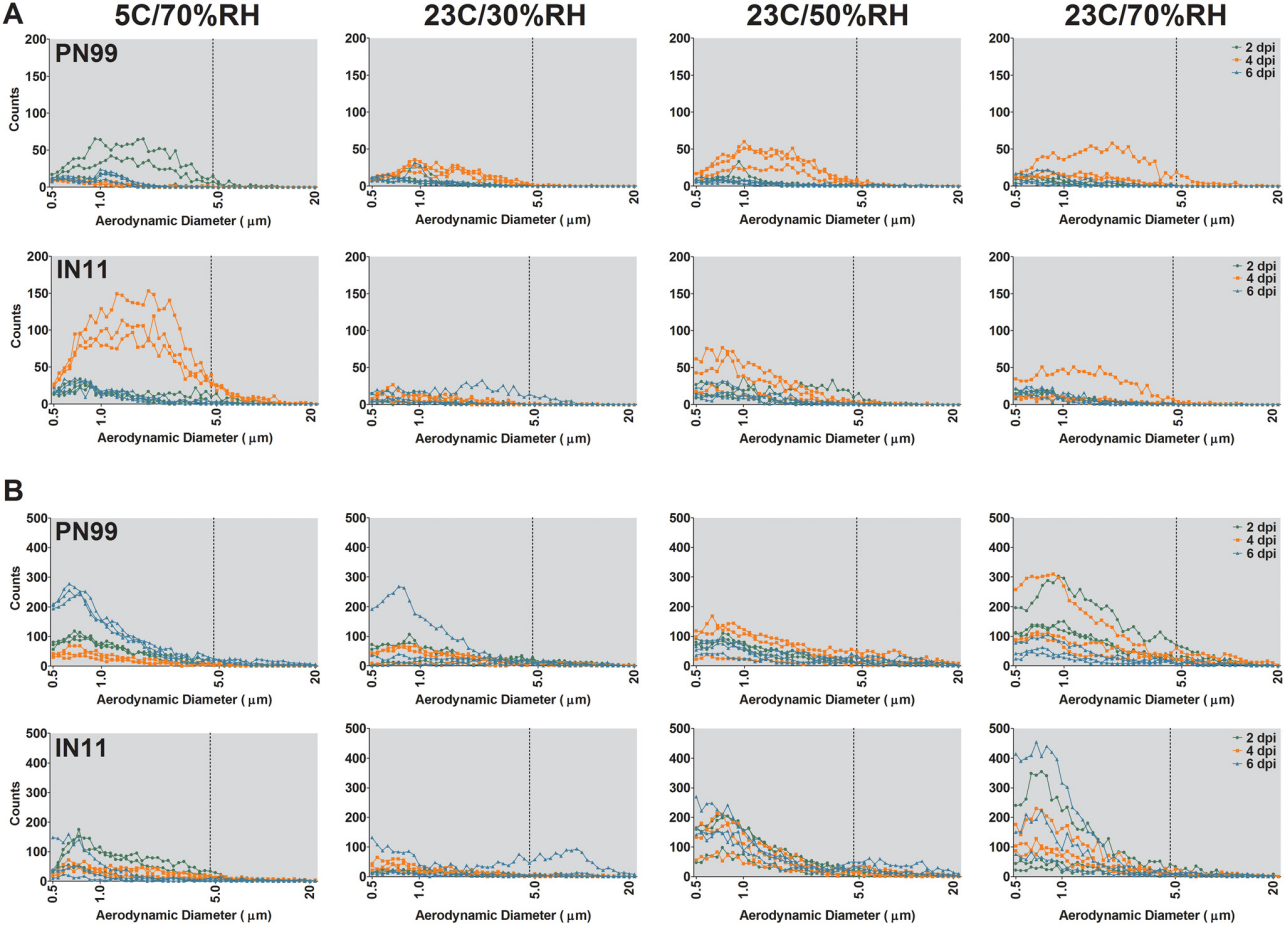
Size distribution of aerosols exhaled from influenza virus infected ferrets. Aerosol particle counts were measured for individual ferrets, n = 3, collected during 15 minutes of normal breathing (A) or 5 minutes of sneezing stimulation (B) on 2, 4 and 6 dpi. Ferrets were housed under controlled environmental conditions as indicated. Aerosol particles within the range of 0.5μm to 20 μm in size are shown with key sizes noted on the *x* axis and a broken vertical line denoting the respirable particle fraction at <5 μm.

Respiratory secretions exhaled into the air serve as the vehicle for influenza viruses to transmit between hosts in the absence of direct or indirect contact. To assess the effects of temperature and humidity on the size distribution of aerosols exhaled from ferrets infected by PN99 or IN11 virus, an APS was used to measure the aerodynamic diameter of particles present in aerosol samples collected on 2, 4 and 6 dpi from ferrets housed at each controlled environmental condition. Aerosol particle counts in exhaled breath were highly variable among ferrets but, for all animals, peaked in the respirable size range (Fig 3). During 15 minutes of normal breathing, aerosol particle counts were highest 4 dpi; significance at this time point was found among all ferret groups (p<0.0001) except PN99 virus at 5°C/70%RH and IN11 virus at 23°C/30%RH (Fig 3A). Overall, particle counts were lowest at the lowest RH condition (23°C/30%RH) (p<0.0001) which is probably because a greater proportion of the aerosol particles are <0.5 μm, below the detection limit of the APS, due to evaporation. During 5 minutes of sneezing stimulation, much more variability was observed between ferrets and between conditions (Fig 3B). Because aerosols were collected for size distribution analysis on days 2, 4 and 6 post-inoculation and nasal wash samples were collected on days 1, 3 and 5 post-inoculation, the kinetics of increased aerosol shedding cannot be linked to the time point at which transmission was observed for each animal. In fact, the highest numbers of aerosol particle counts were measured during normal breathing on 4 dpi in the IN11 virus group housed at 5C/70%RH (Fig 3A) and no transmission was observed in these animals (Fig 2B, Table 1). Particle counts in sneezing samples were highest at 23C/70%RH for both virus groups but transmission occurred among 67% of ferret pairs at this setting.

**Fig 4:**
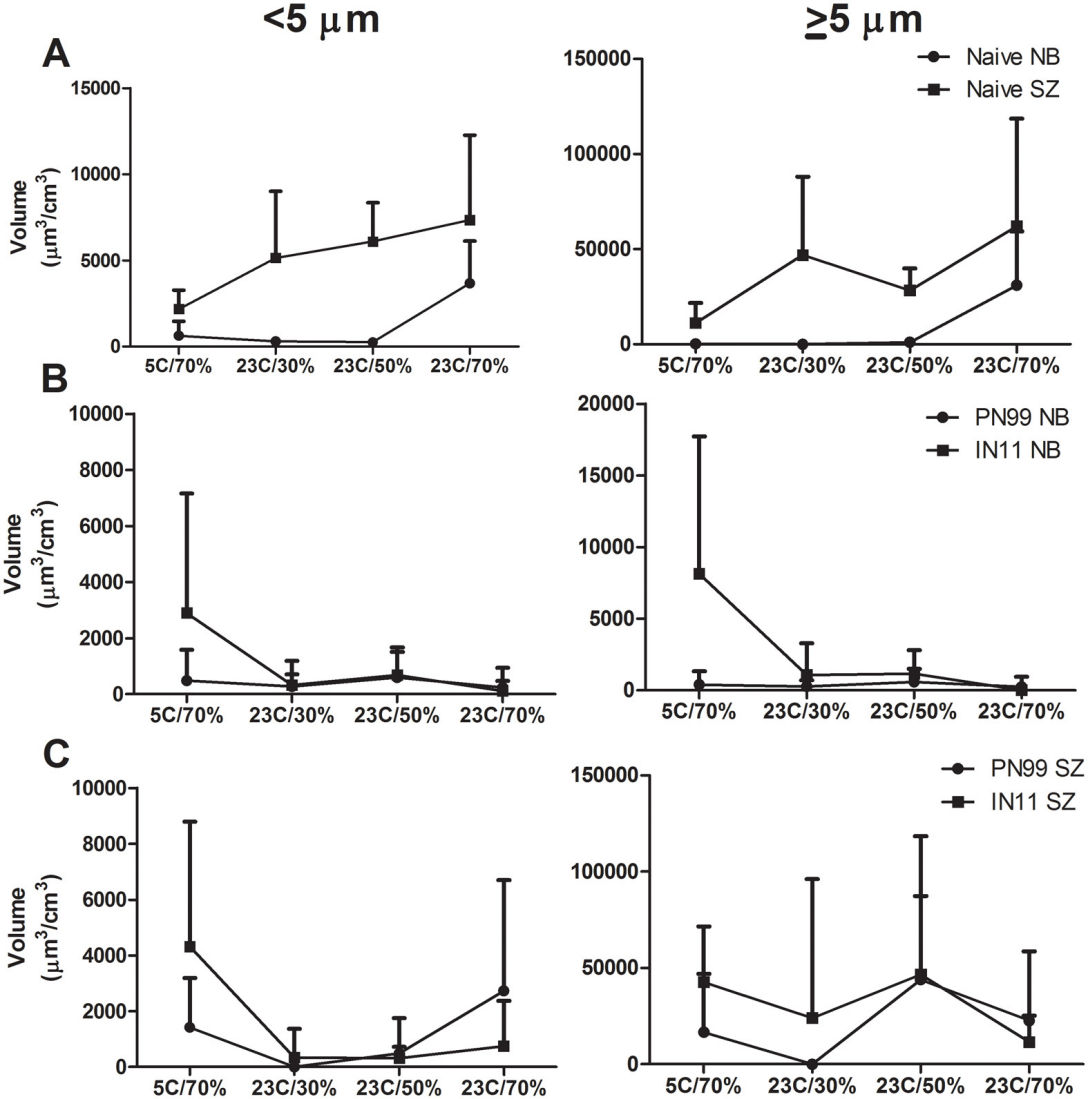
Volume of aerosols exhaled by naïve and influenza virus inoculated ferrets. Aerosol volumes were measured from ferrets during 15 minutes of normal breathing (NB) or 5 minutes of sneezing stimulation (SZ). Data collected from naïve animals, n = 6 (A), and data collected from inoculated animals (normalized to each ferret’s naïve level of aerosol shedding), n = 3, on 2, 4 and 6 dpi were combined and compared between PN99 and IN11 virus groups for aerosols <5 μm and ≥5 μm (B,C). Ferrets were housed under controlled environmental conditions as indicated. Data are presented + standard deviation.

To further compare aerosol shedding among environmental conditions, the volume of aerosols <5 μm and ≥5 μm exhaled by naïve animals was measured and, in general, increased with AH (Fig 4A). Volume data from inoculated animals at all time-points after inoculation were normalized to the respective ferret’s naïve level of aerosol shedding at each environmental setting and were compared between viruses (Fig 4B and 4C). Normal breathing sampling showed similar volumes of exhaled aerosols between PN99 and IN11 viruses with the exception of IN11 virus-inoculated ferrets housed at 5°C/70%RH for both aerosol particle size ranges (<5 μm and ≥5 μm) (Fig 4B). This increase was primarily represented in the sample collected at 4 dpi (Fig 3A). Sneezing samples were more variable but still followed a similar trend between virus groups (Fig 4C). These data indicate that the size distribution of aerosols exhaled by inoculated ferrets did not differ consistently between the PN99 and IN11 virus groups and that the volume of aerosols exhaled by naïve animals generally increased with increasing AH. The overall volume of aerosols collected from ferrets did not appear to be associated with the frequency of transmission events among ferrets housed at the respective environmental settings.

**Fig 5:**
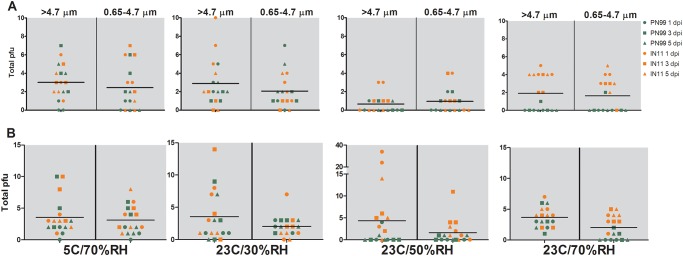
Influenza virus detection in aerosol samples exhaled by infected ferrets. Three ferrets each were housed under the designated environmental conditions and were presented with 10^3.8^–10^5.5^ pfu of PN99 (green) or IN11 (orange) virus by aerosol inhalation. On 1, 3 and 5 dpi, aerosol samples were collected from ferrets for 15 minutes of normal breathing (A) and 5 minutes of sneezing stimulation (B) and were segregated based on size (0.65–4.7 μm or >4.7 μm) and then assayed for the presence of infectious influenza virus. Total plaque forming units (pfu) from individual ferrets, n = 3, is shown with the grand mean for each sampling condition.

**Fig 6:**
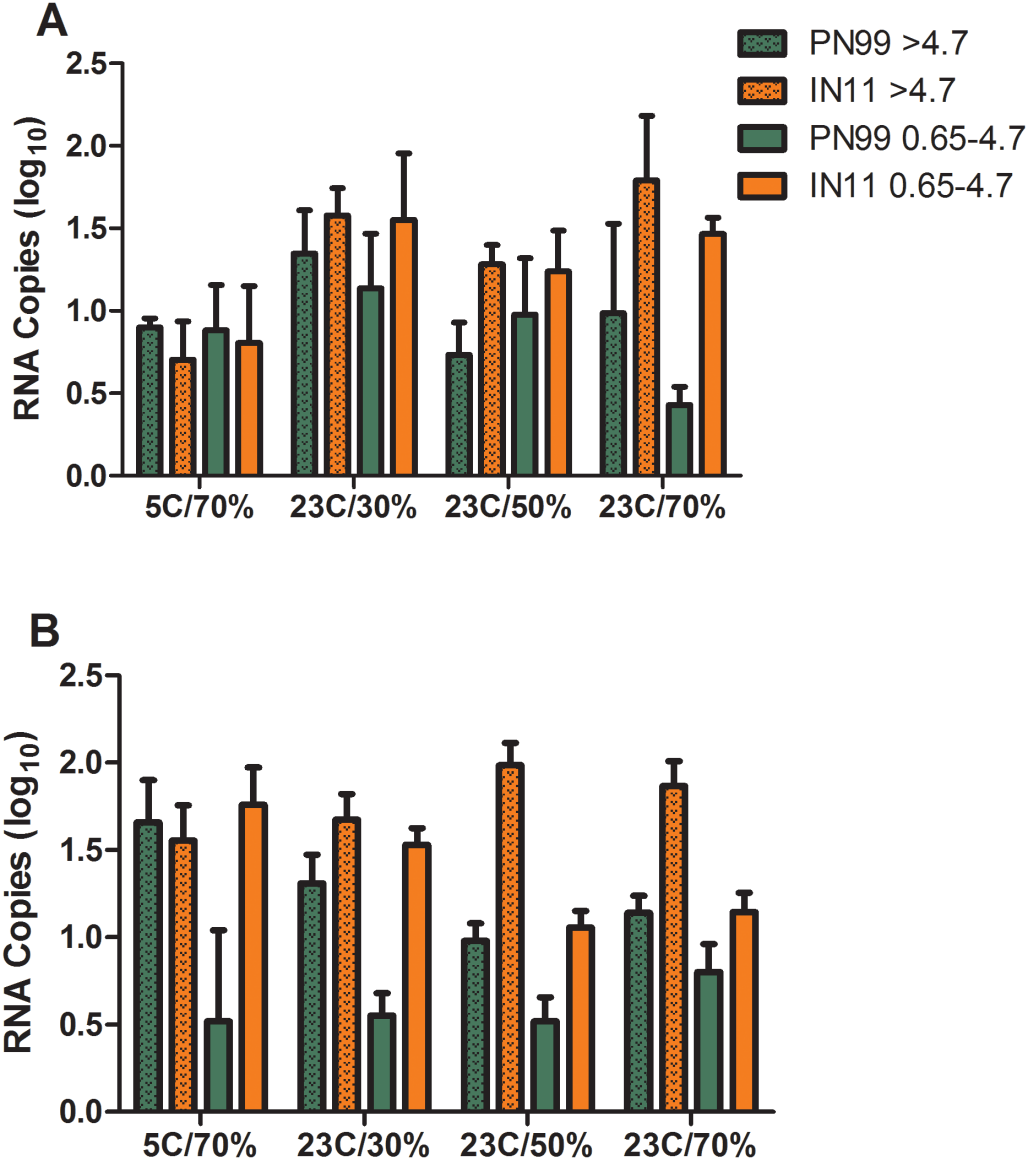
Influenza virus RNA detection in aerosol samples exhaled by infected ferrets. Three ferrets each were housed under the designated environmental conditions and were presented with 10^3.8^–10^5.5^ pfu of PN99 (green) or IN11 (orange) virus by aerosol inhalation. On 1, 3 and 5 dpi, aerosol samples were collected from ferrets for 15 minutes of normal breathing (A) and 5 minutes of sneezing stimulation (B) and were segregated based on size (0.65–4.7 μm or >4.7 μm) and then assayed for the presence of influenza virus RNA copies. RNA copy data from the three time points are combined and presented +SEM for each group.

Any successful aerosol transmission event requires that infectious virus pass through the air from a contagious host to a susceptible one. Using methods designed to maintain the viability of infectious virus in aerosols, sampling of the exhaled breath of ferrets was performed and comparisons were made among the amounts of infectious virus exhaled by influenza virus infected ferrets housed under the controlled environmental conditions. Aerosols were collected for 15 minutes of normal breathing and 5 minutes of sneezing and separated into two size ranges (>4.7 μm and 0.65–4.7 μm) on 1, 3 and 5 dpi and then quantified for influenza virus by plaque assay as well as real-time RT-PCR. Infectious virus detected at any time point and in either size range in normal breathing samples was ≤10 pfu (Fig 5A) and in sneezing samples was ≤34 pfu (Fig 5B). The overall normal breathing grand mean values were lowest at 23°C/50%RH (Fig 5A) while other conditions were similar but slightly higher, and little difference was observed between the two size ranges (<4.7 μm and ≥4.7 μm). With few exceptions, the levels of IN11 virus measured were higher compared to PN99 virus at the respective housing conditions, similar to NW titer comparison results (Fig 2). Likewise, in most cases, IN11 virus RNA levels were higher in these samples compared to PN99 virus. This was most evident in the higher humidity conditions during normal breathing (Fig 6A) and nearly all of the sneezing samples (Fig 6B).

**Fig 7:**
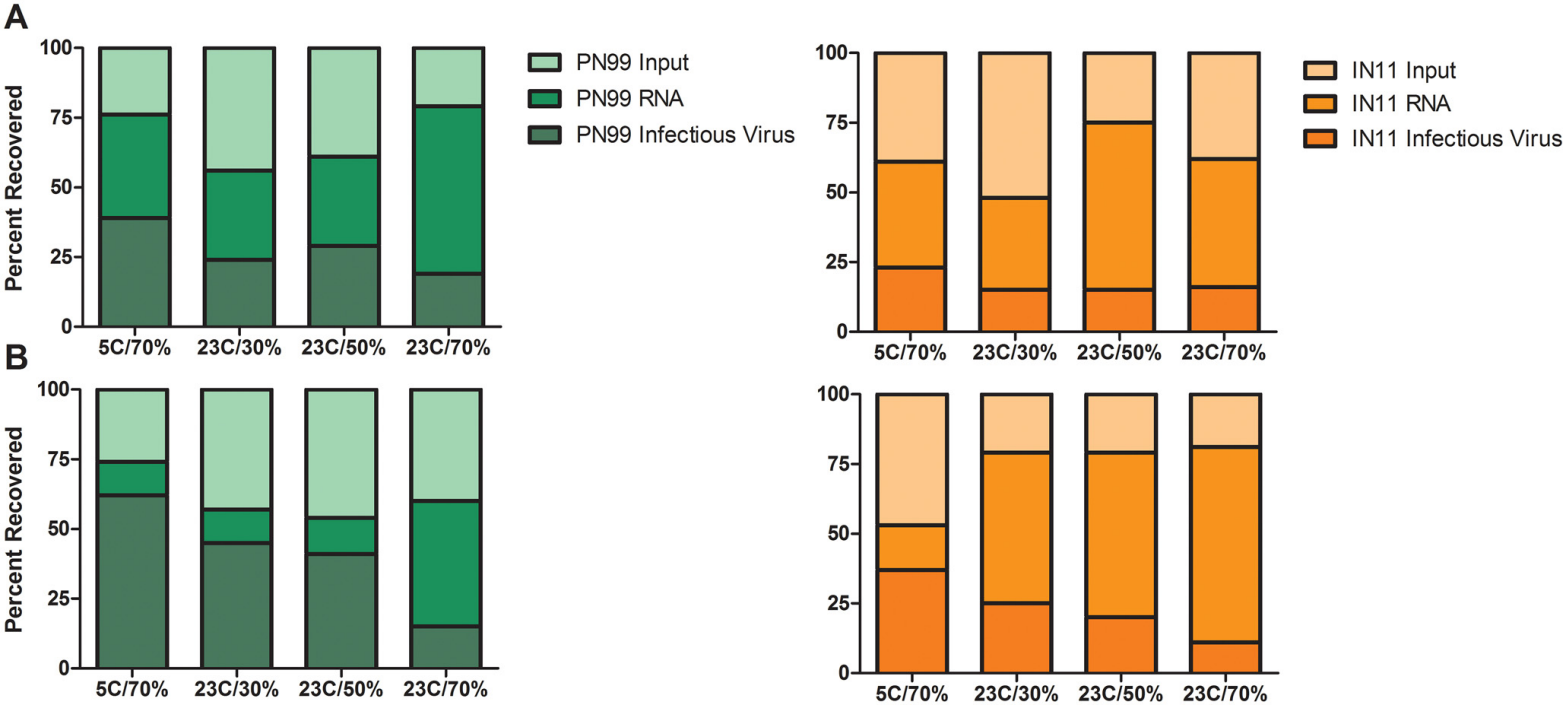
Recovery rates of influenza virus subjected to the aerosol collection procedure. Prepared impactor plates were spiked with 10^2^–10^3^ pfu of PN99 (green) or IN11 (orange) influenza virus and were placed at a designated environmental condition as shown on the x-axis while air was pulled through them for 15 minutes (A) or 5 minutes (B). Experiments were performed in duplicate and the percentage of recovered RNA was determined by real time RT-PCR using M gene primers and infectious virus recovery was based on plaque assays. Each shaded section represents the proportion of the total amount of input virus that was recovered.

**Fig 8:**
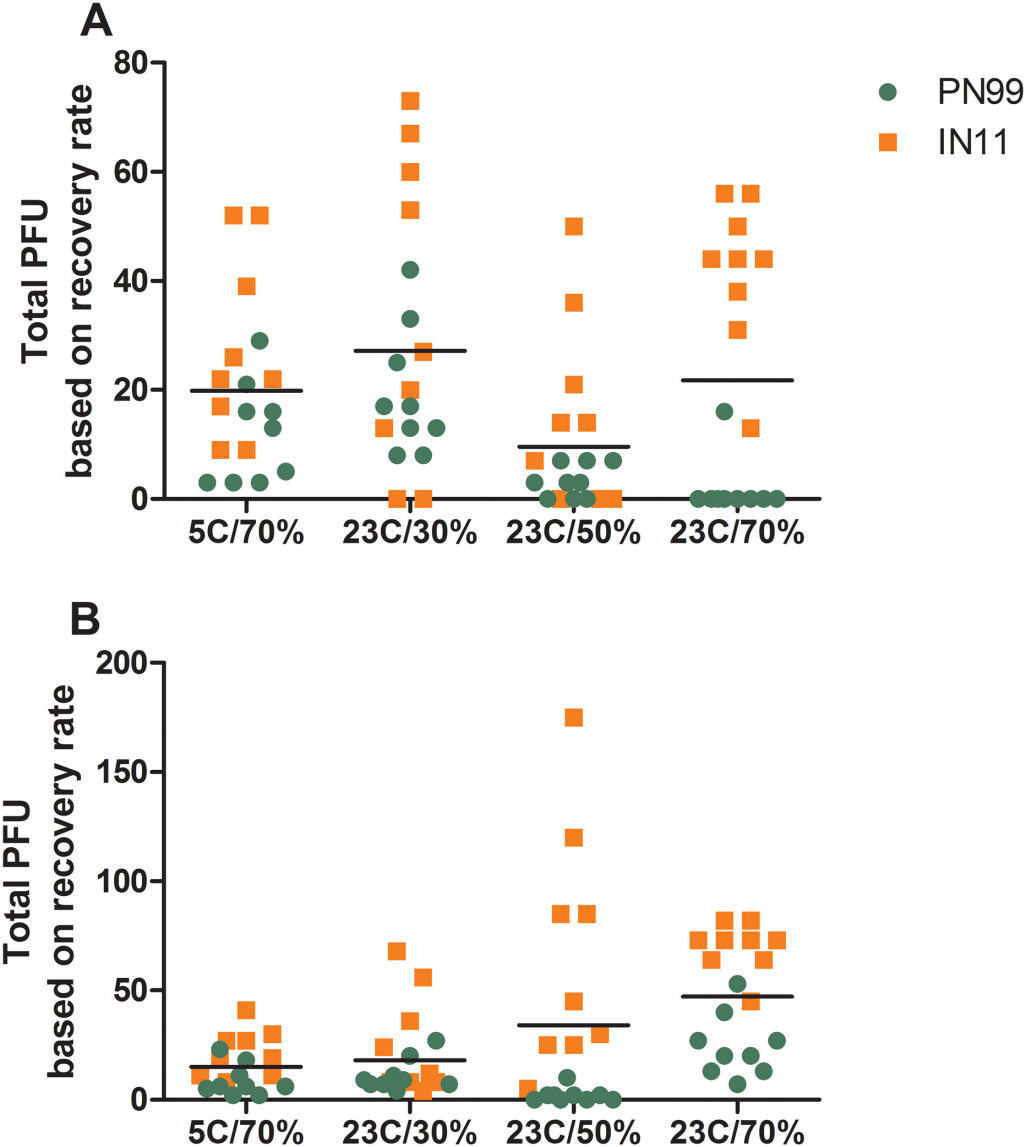
Influenza virus in aerosol samples exhaled by infected ferrets based on recovery rates. Three ferrets each were housed under the designated environmental conditions and were presented with 10^3.8^–10^5.5^ pfu of PN99 (green) or IN11 (orange) virus by aerosol inhalation. Aerosol samples were collected from ferrets on 1, 3, and 5 dpi for 15 minutes of normal breathing (A) and 5 minutes of sneezing stimulation (B). Total plaque forming units (pfu) collected from infected ferrets were normalized based on the recovery rates of known amounts of infectious virus using our aerosol collection procedure. Total pfu exhaled by infected ferrets in both size ranges combined and at each time point are presented. Each dot represents a single animal at a single time point. Scatter dot plots show the distribution of data with the horizontal line representing the grand mean for all samples collected under the designated condition.

The impact of the aerosol collection procedure on virus recovery was tested for each controlled environmental condition by spiking the collection medium on each impactor stage with known quantities of virus. After air was pulled through the impactor for 15 or 5 minutes, to represent normal breathing and sneezing sampling times, respectively, the collection medium was harvested and processed as described for the ferret samples. PN99 virus RNA recovery from either stage and for the 15 and 5 minute collection times ranged from 54 to 79%, similar to IN11 virus RNA recovery which was 47 to 81% (Fig 7). Infectious virus recovery was somewhat more variable at 15 to 62% for PN99 virus and 11 to 37% for IN11 virus. While the recovery rates of RNA illustrate the physical loss of material due to the collection procedure, the loss of infectious virus account for the additional loss due to the inactivation of virus during the collection procedure. However, because virus was applied directly to the gelatin medium, these recovery rates do not necessarily reflect virus susceptibility to the environmental conditions while in an aerosol state but do serve as a means to more accurately estimate the levels of infectious virus exhaled by ferrets during the sampling sessions. Based on these recovery rates, the total amounts (<4.7 μm and ≥4.7 μm combined) of infectious virus exhaled by individual animals during 15 minutes of normal breathing peaked between 7 and 42 pfu for PN99 virus and between 50 and 73 pfu for IN11 virus for all of the environmental conditions tested (Fig 8A). Comparison of mean values between PN99 and IN11 viruses revealed that during normal breathing, regardless of the environmental condition, IN11 yielded greater levels of infectious virus (p = 0.001). Overall, the levels of infectious influenza virus were significantly lower at 23°C/50%RH compared to all other groups (p<0.0001) and 23°C/30%RH was significantly higher than all other groups (p<0.01). As expected, sneezing samples were much more variable, but IN11 virus levels were again higher (Fig 8B). These findings support those derived from NW titer comparisons and confirm that, although the overall amount of IN11 virus being exhaled was greater than PN99 virus, no single environmental condition we chose for housing animals had a significant influence on virus shedding and ultimately on transmission for one virus compared to the other. However, collectively, influenza virus was exhaled during normal breathing in the greatest amount at the 23°C/30%RH condition and the lowest amount at the 23°C/50%RH condition, the same environmental settings at which RD transmission was most and least frequent, respectively.

## Discussion

The influence that temperature and humidity have on aerosolized influenza virus and the consequence for transmission remain poorly understood despite the potential impact on the seasonality of influenza epidemics in the temperate regions of the world. Climatic conditions in tropical regions of the world represent a different set of parameters affecting transmission, and furthermore, routes of transmission not involving airborne virus (e.g. direct or indirect contact) demonstrate the complexity of global influenza virus transmission and the multitude of potential mechanisms at play each impacted differently by environmental conditions. Here, we evaluated the transmissibility of two H3N2 influenza viruses (PN99 and IN11) using ferrets housed under diverse environmental conditions and concurrently evaluated the aerosol shedding profiles of infected animals. We compared the two virus groups to each other and then combined them to identify common differences at each environmental condition. Higher levels of IN11 virus were measured in ferret nasal washes and in exhaled aerosol samples compared to PN99 virus but overall, we found little difference in the transmission capabilities of the two viruses at any of the environmental conditions with transmission most frequently observed at 23°C/30%RH (83%) and least frequently observed at 23°C/50%RH and 5°C/70%RH (17%).

The viruses chosen for this study were selected because one represents a seasonal H3N2 strain (PN99 virus), the other one is a swine-origin H3N2 variant strain (IN11 virus) responsible for a summertime infection [7,25] and both were previously shown to be highly transmissible in the ferret model under normal laboratory conditions [26–28]. Since 2011, H3N2v viruses that possess the M gene segment from the A/(H1N1)pdm09 virus have been isolated from humans and have resulted in over 300 cases in the U.S., primarily in the summer months and in children <10 years of age with a recent history of exposure to pigs [9]. In the years preceding the 2009 H1N1 pandemic, swine-origin H1N1 viruses also caused sporadic infections in young individuals at different times of the year, including the summer [4,5]. This prophetic prelude to the 2009 pandemic stresses the importance of improving our understanding of the transmissibility of H3N2v viruses under different environmental conditions. Epidemiological data suggest that the summertime pattern of H3N2v virus infections is associated with the opportunistic occasion of agricultural fairs that often bring people and pigs together [9]. Individuals 10 years of age and older generally possess some cross-protective immunity to H3N2 viruses providing an explanation for why most of the cases were reported in children under this age [29,30]. However, because the H3N2v human infections were mainly reported during the summer months, comparisons were made in the current study between PN99 and IN11 viruses to explore the potential for enhanced H3N2v virus transmission in a warm, humid environment compared to a virus known to exhibit typical seasonal infection patterns. Although higher levels of H3N2v virus were detected in nasal washes and aerosols exhaled by ferrets housed at the 23°C/70%RH setting compared to the seasonal H3N2 virus (PN99), no difference in the frequency of transmission was observed for the virus pair, indicating that this H3N2v virus has no enhanced ability to transmit under these conditions. In a previous study using the guinea pig model, transmission of PN99 influenza virus through the air was eliminated when animals were housed at 30°C [31] but it is unclear whether a difference between IN11 and PN99 virus transmissibility would reveal itself under warmer housing conditions in the ferret model. Ferrets are naturally cold weather animals and do not tolerate exposure to extremely warm conditions (≥26°C) for prolonged periods of time; they are optimally housed at temperatures of 4–18°C and 40–65% RH [32]. Therefore, we chose housing conditions with this in mind and found that none of our conditions (5°C/70%RH, 23°C/30%RH, 23°C/50%RH, 23°C/70%RH) resulted in substantial morbidity in uninfected ferrets.

Any successful transmission event through the air requires that virus pass from an infected host to a susceptible one while maintaining infectivity. As this occurs, aerosols are released from the warm, humid environment of a respiratory tract, through the ambient conditions outside of the host and eventually reach the warm, humid environment of a recipient’s respiratory tract. Depending on the ambient conditions and the solute composition of the aerosol, evaporation and condensation processes may have deleterious effects on any virus present in the aerosols [16]. Using coughing and breathing simulators in a recreated examination room, researchers demonstrated that influenza virus infectivity is markedly reduced at ≥43%RH [17]. Similarly, the infectivity of influenza virus aerosolized at 40%RH was significantly impacted [33]. Transmission studies using guinea pigs revealed reduced RD transmission capabilities of influenza virus at 50%RH [18]. In the current study using the ferret model, reduced RD transmission was also observed (down to 17% with both virus groups combined) at 50%RH. Further, we observed lower levels of infectious virus present in aerosols exhaled by infected animals but not in nasal wash samples collected from ferrets housed at 23°C/50%RH demonstrating the added advantage of including the analysis of aerosol samples as a parameter in influenza transmission studies. At 70%RH (with a consistent 23°C setting), RD transmission frequency was somewhat improved to 67% but was most efficient at the low humidity, 30%RH, setting. Under cold conditions (5°C) and 70%RH, RD transmission was rare, occurring between 17% of ferret pairs. However, the levels of infectious virus measured in aerosols exhaled by ferrets at 5°C/70%RH were not reduced, unlike our observations of animals housed at 23°C/50%RH. This disparity may be explained by the fact that water vapor released into a cold, humid environment can quickly convert to a liquid [34]. In other words, we can speculate that as the animal exhales air from the warm, humid environment of their respiratory tract, it encounters the colder ambient air (5°C/70%RH), which may cause some of the exhaled aerosol particles to essentially “grow” by condensation, thereby increasing the settling velocity of those exhaled droplets [34]. Thus, the frequency of transmission events occurring through the air at 5°C/70%RH is reduced but aerosols collected immediately after being exhaled by the animal do not show any reduction in the levels of infectious virus. Experiments conducted in guinea pigs housed at 5°C and 65%RH or 80%RH displayed successful RD transmission 50% of the time but directional airflow used during those experiments may have assisted the transport of virus-laden aerosols from infected animals to their uninfected counterparts [18]. However, both guinea pig and ferret studies concur that RD transmission is most efficient in low RH environments. Mechanisms have been proposed to explain the loss of infectivity of influenza virus aerosolized at mid-range RH settings (reviewed in [14]). As water evaporates from aerosol particles, the solute components become more concentrated which can have damaging effects on influenza virus being carried within those aerosols. At high humidity environments, evaporation is minimal, but at low humidity environments, below the efflorescence RH (~50%RH), the solute components crystallize rendering the virus intact and viable [16]. This may be the reason why we observed reduced exhaled infectious influenza virus and ultimately reduced frequency of RD transmission in ferrets housed at 23°C/50%RH.

Detection of influenza virus in exhaled aerosols has been largely limited to the detection of viral genetic material via RT-PCR with few reporting infectious virus detection in humans [12,13] or laboratory animals [35,36]. We previously developed a method to collect size-segregated, exhaled aerosols from ferrets using a cascade impactor and measure the total amount of infectious influenza virus present [24]. Those experiments were conducted under normal laboratory conditions (21°C/30%RH) without the use of an environmental chamber, and we found similar results as reported here for ferrets housed at the 23°C/30%RH setting, including efficient RD transmission of PN99 and IN11 viruses [26–28]. Because there is unavoidable loss of viral material during our aerosol collection and processing procedure, recovery rates were derived to provide improved estimations of the amounts of virus in the exhaled breath of infected ferrets. Comparison of these estimated levels of virus were as follows: 23°C/30%RH > 23°C/70%RH > 5°C/70%RH > 23°C/50%RH, similar to the overall frequency of RD transmission (83%, 67%, 17%, 17%, respectively). Unlike normal breathing samples, the levels of influenza virus measured at each condition during sneezing sessions was not consistent with the frequency of transmission events (i.e. the greatest amount was measured at 23°C/50%RH), suggesting that the virus released during normal breathing may be more instrumental in influenza transmission through the air. It has been suggested that although sneezing and coughing events send more aerosols into the air per maneuver compared to normal breathing, over the course of an entire day, significantly more aerosols will be released during normal breathing because breathing is continuous [37]. Furthermore, the substantial heterogeneity in aerosols generated by ferrets during sneezing usually confounds analysis of data obtained from these highly variable samples. Despite these challenges, increased sneezing is routinely reported in ferrets infected by influenza viruses [26,38,39] so the contribution of virus-laden aerosols expelled by ferrets during sneezing to transmission cannot be ruled out for this animal model.

In the temperate regions of the world, people spend the vast majority of their time indoors, especially during the winter months where the environment is typically maintained at a warm temperature and low RH [1]. This scenario sets the stage for influenza virus transmission through the air by providing favorable conditions for maintaining virus viability in aerosols while, due to high evaporation rates, creating a large fraction of respirable particles capable of prolonged suspension in the air [1]. Improving our understanding of how environmental conditions modulate influenza virus infectivity and transmission could help reveal mechanisms that can be exploited to develop novel prevention strategies that better protect people from influenza virus infection. There are inherent limitations in the techniques used to evaluate the role of bioaerosols in influenza virus transmission. Most notably, we are challenged with isolating infectious influenza virus from aerosol samples collected from the exhaled breath of infected hosts at levels high enough for meaningful evaluation. Collection times typically represent brief snapshots within the overall kinetics of virus shedding. Unfortunately, increasing collection times often compromise aerosol collection efficiency and the preservation of virus infectivity. Innovative strategies in multiple laboratories are being pursued to improve these techniques [12,35,40,41]. Additionally, even the most suitable animal model will not always accurately represent the presentation of influenza virus infection in humans and ethical responsibilities necessitate the use of a minimum number of animals. Nevertheless, knowledge gained through controlled animal studies using both virological and aerobiological techniques will ultimately further our understanding of influenza virus infection and transmission in the diverse environmental conditions experienced throughout the year and around the world.
